# The Impact of COVID-19 on Interventional Radiology Practice Worldwide: Results from a Global Survey

**DOI:** 10.1007/s00270-022-03090-6

**Published:** 2022-03-11

**Authors:** F. Gomez, P. Reimer, P. L. Pereira, C. Bent, R. L. Cazzato, M. Das, A. Diamantopoulos, B. Zeka, N. Kaufmann, G. Makris

**Affiliations:** 1 Interventional Radiology, Hospital Clinic de Barcelona, Barcelona, Spain; 2 Interventional Radiology, Antoni van Leewenhoek-Netherlands Cancer Institute, Amsterdam, The Netherlands; 3 Diagnostic and Interventional Radiology, Klinikum Karlsruhe, Karlsruhe, Germany; 4 Radiology, Minimally Invasive Therapies and Nuclearmedicine, SLK-Clinics GmbH Heilbronn, Ruprecht-Karls-University Heidelberg, Heilbronn, Germany; 5 Interventional Radiology, University Hospitals Dorset, Bournemouth, UK; 6 Interventional Radiology, University Hospital of Strasbourg, Strasbourg, France; 7 Department of Diagnostic and Interventional Radiology, Helios Klinikum Duisburg, Duisburg, Germany; 8 Department of Interventional Radiology, Guy’s and St. Thomas’ NHS Foundation Trust, London, UK; 9 Faculty of Life Sciences and Medicine, School of Biomedical Engineering and Imaging Sciences, Kings College London, London, UK; 10 Clinical Research Department, Cardiovascular and Interventional Radiological Society of Europe, Neutorgasse 9, 1010 Vienna, Austria

**Keywords:** COVID-19, Coronavirus, Pandemic, Second wave, IR, Interventional radiology, IR services

## Abstract

**Background:**

The COVID-19 pandemic had an unprecedented impact on clinical practice and healthcare professionals. We aimed to assess how interventional radiology services (IR services) were impacted by the pandemic and describe adaptations to services and working patterns across the first two waves.

**Methods:**

An anonymous six-part survey created using an online service was distributed as a single-use web link to 7125 members of the Cardiovascular and Interventional Radiological Society of Europe via email. Out of 450 respondents, 327 who completed the survey at least partially including 278 who completed the full survey were included into the analysis.

**Results:**

Interventional radiologists (IRs) reported that the overall workload decreased a lot (18%) or mildly (36%) or remained stable (29%), and research activities were often delayed (30% in most/all projects, 33% in some projects). Extreme concerns about the health of families, patients and general public were reported by 43%, 34% and 40%, respectively, and 29% reported having experienced significant stress (25% quite a bit; 23% somewhat). Compared to the first wave, significant differences were seen regarding changes to working patterns, effect on emergency work, outpatient and day-case services in the second wave. A total of 59% of respondents felt that their organisation was better prepared for a third wave. A total of 19% and 39% reported that the changes implemented would be continued or potentially continued on a long-term basis.

**Conclusion:**

While the COVID-19 pandemic has negatively affected IR services in terms of workload, research activity and emotional burden, IRs seem to have improved the own perception of adaptation and preparation for further waves of the pandemic.

**Supplementary Information:**

The online version contains supplementary material available at 10.1007/s00270-022-03090-6.

## Introduction

After the start of the COVID-19 pandemic in March 2020, interventional radiological societies all over the world published many guidelines on how to continue services for urgent procedures while considering cross-contamination and patient, as well as staff safety. Benefitting from the experiences from SARS (severe acute respiratory syndrome) and MERS (Middle East respiratory syndrome), temporal and spatial segregation of high-risk patients, use of personal protective equipment (PPE) and segregation of teams were identified as important measures to avert the spread of the virus. Based on key publications, CIRSE has published a checklist for preparing interventional radiology services (IR services) for COVID-19 [1]. Recommendations also suggested the postponement of both non-urgent and elective procedures [2–7]. The published literature showed that during the first wave, the overall number of procedures performed by interventional radiologists (IRs) decreased by 16–62%, out-of-clinics hours and stress increased, and the number of outpatient cases was affected [8–12].

Surveys collected in the UK and Canada during the first wave confirmed the reduction in IR services, particularly elective treatments and reported absence of training on the use of PPE [13–15]. As the second wave began, further postponement and delays in provisions for IR services were not regarded as a sustainable solution, and many IRs worried about the negative effect on the wellbeing of patients [16–19].

Our survey aimed to assess how interventional radiology departments across the world adapted in the face of the COVID-19 pandemic. More specifically, we aimed to gain insight into the role of minimally invasive therapies in patient management as well as to assess the workload of IRs in this pandemic. Finally, we attempted to assess which measures could be implemented to facilitate future transitions between standard care and pandemic emergency care.

## Methods

### Survey Design and Distribution

An anonymous electronic survey (Alchemer LLC, Louisville, USA) was designed to capture the impact of the COVID-19 pandemic on workload, service delivery as well as on people and teams. The survey contained 78 questions in total. The study and questionnaire were reviewed and approved by the CIRSE scientific committee. The full questionnaire is available in the supplementary document 1.

**Fig. 1:**
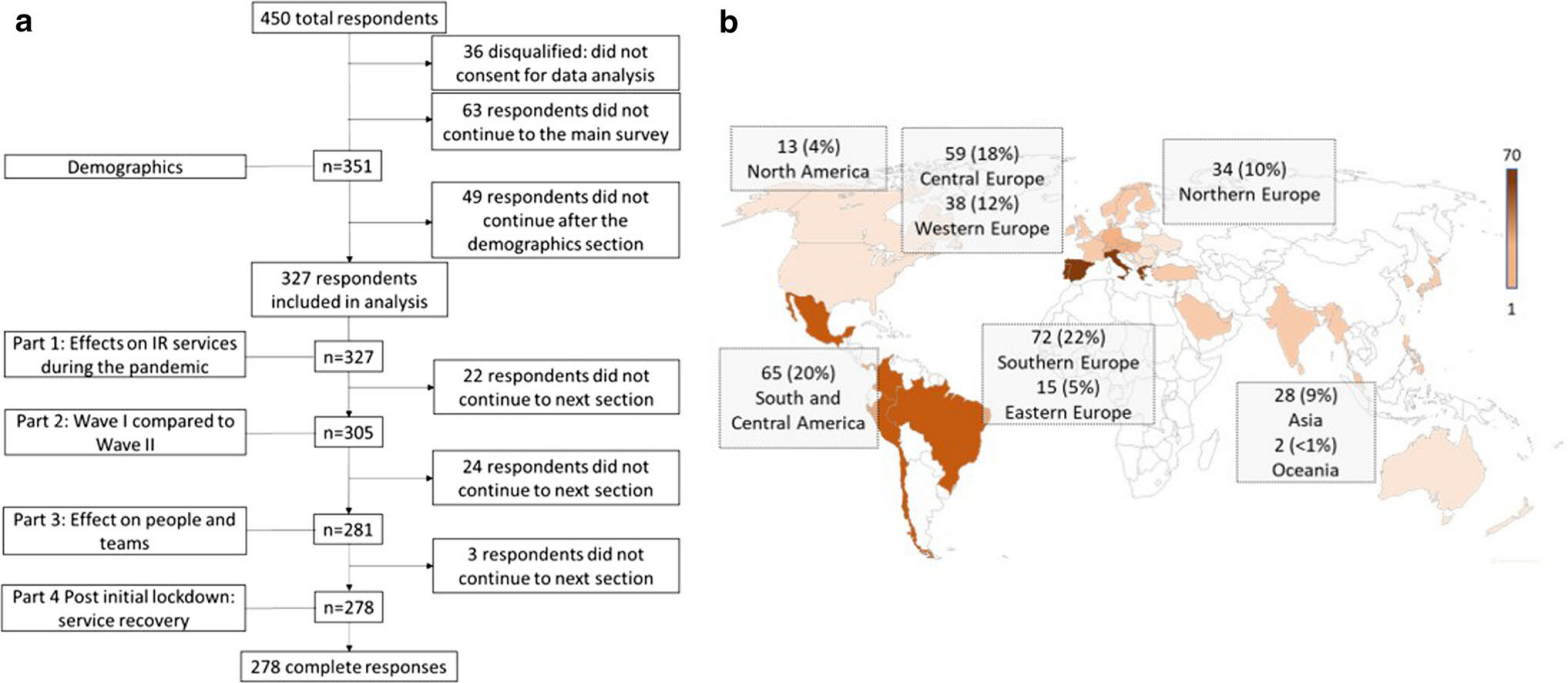
Flowchart summarising number of respondents per survey part (**a**). World map indicating number of respondents (percentage) per region. The colour code ranges from the highest number of respondents (dark) to the lowest number of respondents (light) (**b**)

The proportion of respondents who completed different parts of the survey is shown in Fig. 1. Three hundred twenty-seven (327) respondents who had completed at least one part were included into the analysis. Two hundred seventy-eight (*n* = 278, 85.01%) out of all included respondents had completed the whole survey. For the analysis of the differences between the first and second wave, respondents were instructed to compare “March 2020–June 2020” as the first wave to “September 2020 onwards” as the second wave of the COVID-19 pandemic, and only complete responses were considered. The survey was circulated via email to 7125 CIRSE members. To maximise responses, reminders via email and via CIRSE e-newsletters were sent out. Additionally, social media posts on multiple platforms were employed to increase the dissemination of the survey. Data were collected between 17 December 2020 and 8 March 2021.

### Statistical Analysis

Data are presented as counts and percentages of responses for tables and as percentages for figures. Significant differences between categorical variables were assessed using Fisher exact test (*p* values ≤ 0.05 were considered significant). Data were analysed and plotted using R Studio under R4.0.0.

## Results

### Demographics

Most (67%) respondents were from Europe or from South and Central America (20%). The majority were employed in tertiary centres (46%), followed by public district general hospitals (23% > 500 beds, 16% < 500 beds) and finally, private hospitals (17%). Forty-three per cent (43%) of respondents were board certified radiologists and 42% had completed interventional radiology training or were specialists in interventional radiology. Eighty-seven per cent (87%) of respondents stated that their department had cared for COVID-19 patients (Table 1).

**Table 1:** Demographic information

Respondents	*n*	%
*Role in department*		
Board certified radiologist	139	43
Completed interventional radiology training/specialist	136	42
Resident	17	5
Interventional radiology fellow in training	13	4
Head/director of interventional radiology/radiology department	9	3
Consultant interventional radiologist	4	1
Chief physician/interventional radiologist	3	1
Radiographer	1	1
*Age group*		
< 35	42	13
35–45	129	39
46–55	99	30
56+	57	17
*Gender*		
Male	270	83
Female	57	17
*Region*		
Central Europe	59	18
Southern Europe	72	22
Northern Europe	34	10
Western Europe	38	12
Eastern Europe	15	5
South America and Central America	65	20
North America	13	4
Asia	28	9
Oceania	2	< 1
Africa	1	< 1
*Institution type*		
Tertiary centre	149	46
Public district general hospital (> 500 beds)	74	23
Public district general hospital (< 500 beds)	52	16
Public district general hospital	8	2
Private hospital	55	17
University hospital	4	1

Interventional radiology service	*n*	%
*Number of interventional radiologists*		
< 5	85	26
5–10	57	17
10+	14	4
*Number of dedicated personnel*		
< 5	14	4
5–10	63	19
10+	79	24
*Department cared for COVID patients*		
Yes	285	87
No	39	12
Unsure	2	1
*Services offered*		
Elective embolization	163	50
Hepatobiliary services	164	50
Interventional oncology	155	47
Endovascular peripheral arterial services	139	43
Endourology services	100	31
Endovascular aortic services	93	29
*Out of hours interventional radiology services*		
Yes 24/7	131	40
Yes, but limited (not 24/7)	27	8
No, only on call	19	6
Unsure	3	1
*Number of dedicated in-patient beds*		
0	135	41
< 5	17	5
5–10	16	6
10+	8	2

### Impact on Services and Workload

**Fig. 2:**
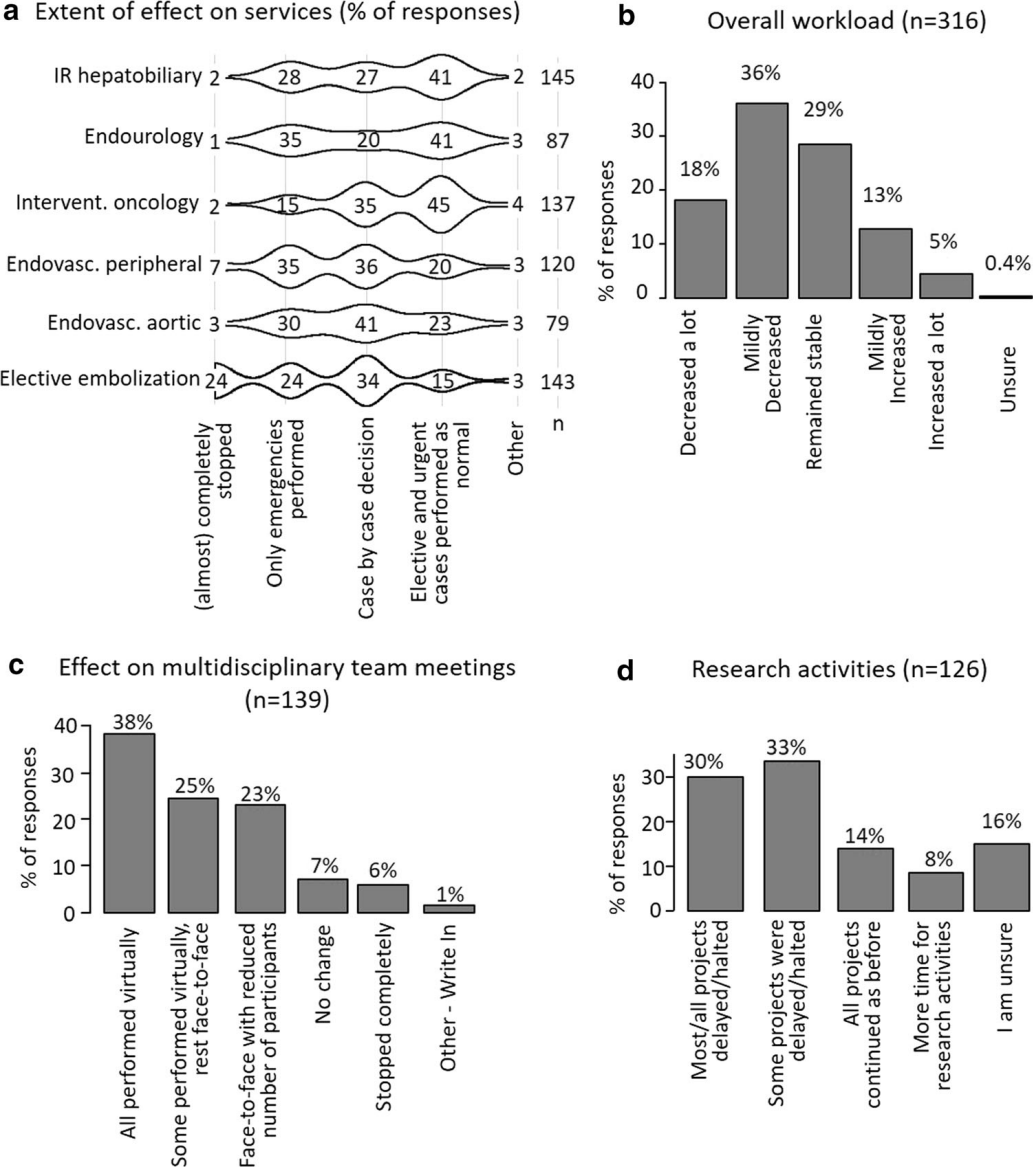
Effect on IR services, work load and staff. Percentage of selections for the type of effect are listed for the respective service (**a**). Bar plots indicating percentage of selections of statements (**b–c**). Violin plots with type of effect on x-axis and services on y-axis

The pandemic was reported to have affected various IR services differently (Fig. 2a). Hepatobiliary, endourology and interventional oncology procedures appeared to be the least affected during the pandemic with 41%, 41% and 45% of the participants, respectively, reporting that they continued offering these services. Peripheral and aortic work and elective embolization procedures such as fibroid or prostate artery embolization were affected more with 20%, 23% and 24% of the participants, respectively, reporting that they continued offering these services. The overall workload generally either decreased (18% a lot, 36% mildly) or remained stable (29%). A total of 18% reported an increase in overall workload (13% mildly increased; 3% increased a lot). When comparing those to responses from IRs who reported no increase, we found significant differences indicating that working hours had been less consolidated, day-case clinics were affected less, and emergency work had increased in volume more (supplementary document 2). As expected, research activity was severely affected with more than 60% of the participants reporting that research projects were either stopped or significantly delayed (Fig. 2b, d).

Multidisciplinary team meetings were either totally (38%) or partly (25%) performed virtually or performed face-to-face with reduction in number of participants (23%) (Fig. 2c).

### Burden on IRs

**Fig. 3:**
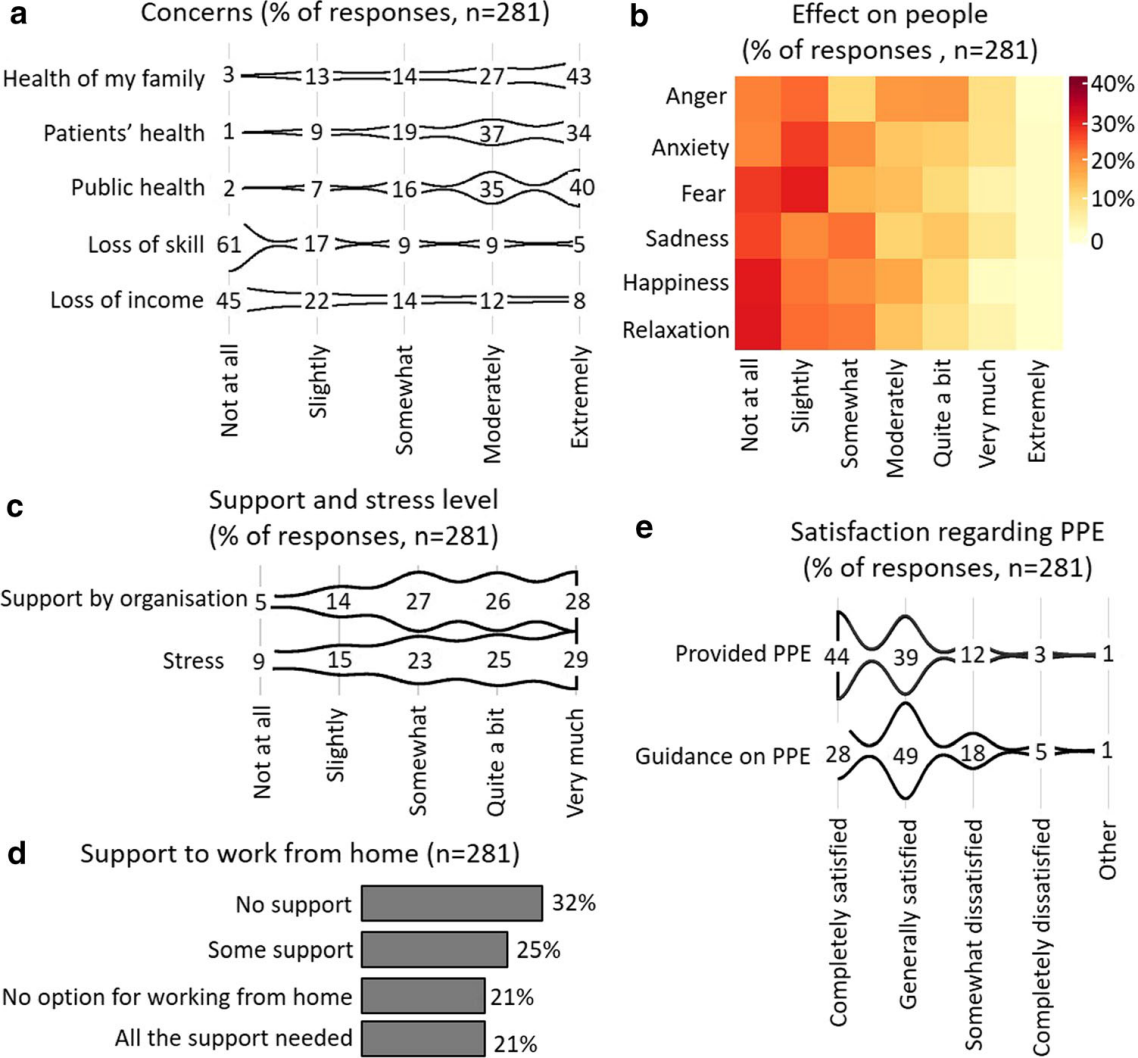
Effect on people and team. Violin plots for extent of effect (**a**, **c**) or satisfaction (**e**) on x-axis and areas on y-axis. Percentage of selections for the extent are listed for the respective areas. Heatmap of effect on people with type of effect on the y-axis and extent of effect on x-axis generated using the R heatmap function with no clustering (**b**). Bar plot indicating percentage of selections of statements (**d**)

While IRs were generally not concerned about loss of skill or income, IRs reported concerns about the health of families, patients and general public (43%, 34% and 40%, respectively, extremely concerned) (Fig. 3a). Approximately, 30% reported feeling slightly fearful or anxious (Fig. 3b).

Despite respondents stating that they were supported by their organisations (28% very much; 26% quite a bit; 27% somewhat), many reported high levels of stress (29% very much; 25% quite a bit; 23% somewhat) (Fig. 2c). IRs were completely or generally satisfied with provided PPE (44% and 39%, respectively) and the guidance on PPE (28% and 49%, respectively) (Fig. 3e) with no statistical difference between tertiary centres, public district general hospitals (> 500 beds), public district general hospitals (< 500 beds) and private hospitals (data not shown).

### Adaptation of Working Patterns and Patient Care

**Fig. 4:**
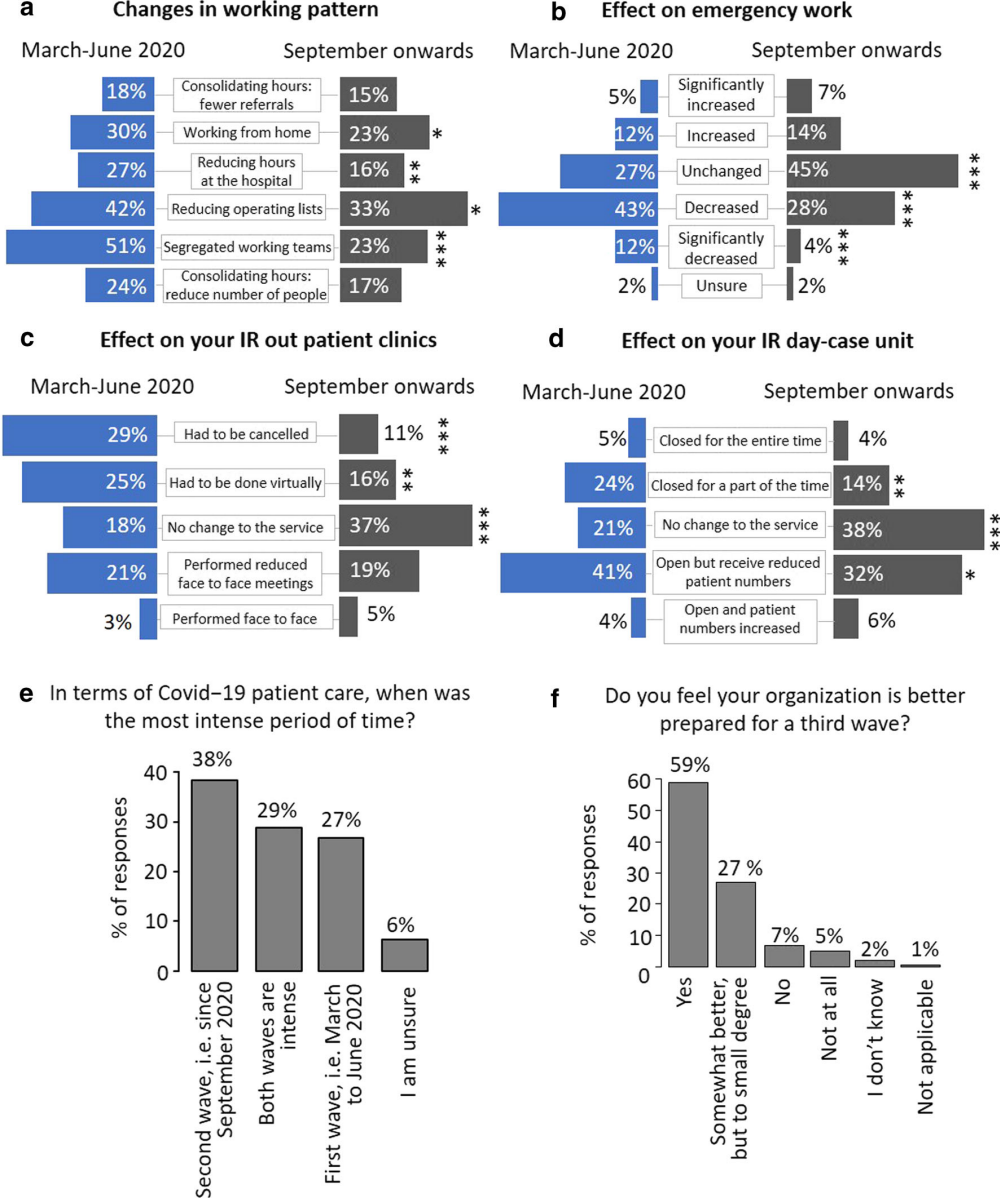
Comparing the first wave of the pandemic to the second wave. Changes in working patterns and effect in emergency work and patient care during the first wave and the second wave of the pandemic. Bar plots indicating % of selections of statements for March to June 2020 (left) and September onwards (right) (**a**–**d**). Bar plot indicating percentage of selections of statements (**e**, **f**). Significant differences between categorical variables were assessed using Fisher exact test (*p ≤ 0.05, **p ≤ 0.01, ***p ≤ 0.001)

Adaptations to accommodate patient and staff safety differed between the first wave (March 2020–June 2020) and the second wave (September 2020 onwards) of the pandemic (Fig. 4). Comparisons of the first wave vs the second wave showed that significantly fewer respondents indicated “working from home” (30 vs 23%, *p* < 0.03), “reducing hours at the hospital” (27 vs 16%. *p* < 0.009), “reducing operating lists” (42 vs 33%, *p* < 0.04) and “segregated working teams” (51 vs 23%, *p* < 0.001) (Fig. 4a). Generally, only a small number of IRs reported to have at least partially been redeployed to other departments but we could observe that this number increased for the second wave of the COVID-19 pandemic (12 vs 35%; data not shown).

When asked about how emergency work had been affected, significantly more respondents reported “unchanged” for the second wave (45 vs 27%, *p* < 0.001), and fewer respondents indicated “decreased” and “significantly decreased” during the second wave of the pandemic (28 vs 43%, *p* < 0.001 and 4 vs 12% *p* < 0.001, respectively) (Fig. 4b). Similarly, regarding outpatient consultations and patient flow through day-case units, more respondents reported “no change to the service” during the second wave (37 vs 18%, *p* < 0.001 and 38 vs 21%, *p* < 0.001, respectively) (Fig. 4c, d). For outpatient clinics, fewer responses for “had to be cancelled” or “had to be done virtually” were given for the second wave (11 vs 29%, *p* < 0.001 and 16 vs 25%, *p* < 0.01), and for day-case units, fewer responses were given for “closed part of the time” and “open but received reduced patient numbers” for the second wave (14 vs 24%, *p* < 0.003 and 32 vs 41%, *p* < 0.04, respectively). Interestingly, though, the second wave of the pandemic was considered more (38%) or equally intense (29%) compared to the first wave (Fig. 4e). Finally, when asked whether the respondents felt that their organisation was better prepared for a third wave, 59% responded with “yes” and 27% with “somewhat better, but to a small degree” (Fig. 4f).

### Post-wave Routines

**Fig. 5:**
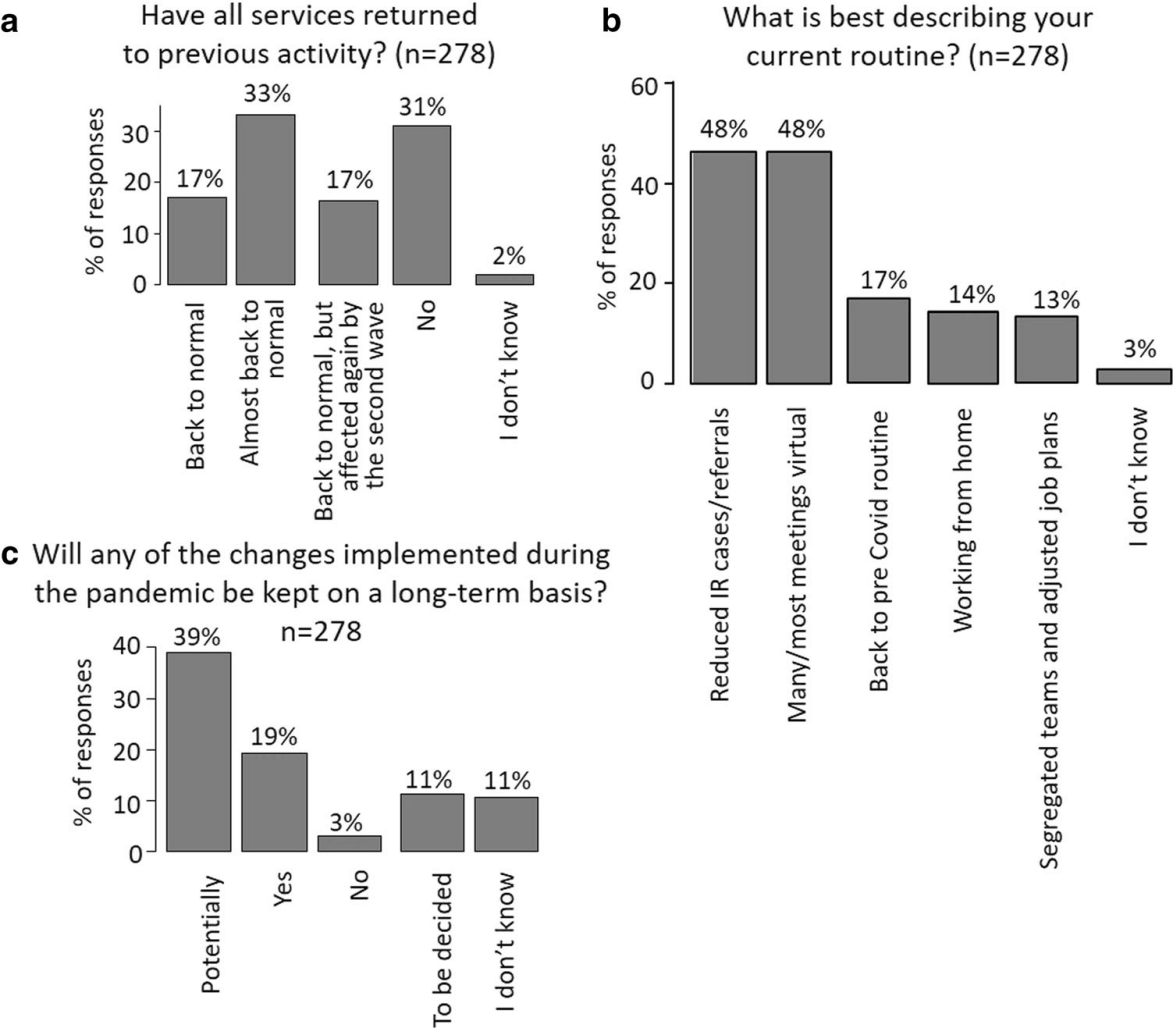
Returning to pre-COVID routine. Bar plots indicating percentage of selections of statements

Following the first wave, 17% and 33% indicated that the services were back to normal or almost back to normal, respectively, 31% reported that services were still affected by the pandemic, and 17% indicated that services had returned to previous activity but were affected again during the second wave (Fig. 5a). Most respondents indicated that cases/referrals were still reduced (48%) and that many/most meetings were still held virtually (48%). A number of IRs reported that the changes that had been implemented during the pandemic would be continued (19%) or potentially continued (39%) on a long-term basis (Fig. 5c).

## Discussion

Research from centres all over the world is confirming the disruptive effect of the COVID-19 pandemic on healthcare [20–22]. The results of our survey showed that there was a general reduction in overall workload, though not all aspects of IR services were affected equally. Hepatobiliary, interventional oncology and endourology procedures did not appear to have been affected as much as endovascular peripheral/aortic procedures and elective embolization. Elective embolization was reported to have stopped completely or almost stopped more often than any other service in question. This illustrates the implementation of the general recommendations to prioritise urgent and oncologic treatments and postpone non-urgent procedures. However, as the second wave followed, it became apparent that long-term adaptations and implementation of routes to provide services safely rather than repeatedly postponing non-urgent care, as well as monitoring the burden on the mental health of IRs and IR service staff, were necessary.

The risk of recurrence and gravity of further waves, combined with mounting pressures secondary to postponed procedures, particularly in the cancer-care setting, has led to worldwide concern and highlighted the need for robust service recovery plans. In survey results from radiologists, 90% of respondents reported reduction in workload and 60% indicated that a workload reduction of over 50% [23]. According to surveys targeting neurosurgery, pancreatic surgery, cardiac surgery or general surgery residents conducted in the first half of 2020, 62–93% of the respondents reported a decrease in cases [24–27]. In fact, for surgical procedures during the pandemic phases, only emergency and essential-elective surgeries (cancer and transplant surgery) are recommended to be performed. Recommendations suggest postponement of non-urgent cases, or conversion to alternative suitable non-operative/minimally invasive procedures [28–30]. As a result, interventional radiology departments are required to be highly adaptive and accommodate this inflow of patients from other medical disciplines. The rigorous discourse of the community might have already enabled initial recovery during the second wave of the pandemic. In order to be able to provide COVID-positive and COVID-negative patients with IR services, structural organisations regarding procedural and transfer logistics were put in place. These included establishing separate routes and rooms for transfer when treating COVID-positive patients, performing bed-side ultrasound-guided procedures and increasing telehealth for outpatient clinics [3, 4, 10, 11, 31–33]. Our data captured differences in how the second wave of the pandemic (September 2020 onwards) was handled compared to the first wave (March 2020–June 2020). During the second wave of the pandemic, IRs reported reduction in operating lists and segregation of working teams less frequently despite the fact that COVID-19 patient care was considered more or equally intense. Likewise, less reduction in emergency work, outpatient clinics and day-case units were reported during the second wave of the pandemic.

Interventional radiology appeared to be well-suited to adapt to times of limited resources regarding hospital beds, anaesthesiologists and staff. Minimally invasive procedures such as radioembolisation and ablation can be optimal treatments for local tumour control while requiring shorter hospital stays [34–36]. Additionally, the use of local anaesthesia or alternative sedation methods to replace general anaesthesia help reduce the length of hospital stays, which, combined with appropriate pre- and postprocedural medications, often allows for outpatient treatment without always requiring the presence of an anaesthesiologist [37–40]. The reduced disruption to working patterns, emergency work and day-case/outpatient units showed that IRs could adapt to the requirements of the pandemic. Beyond being able to return to usual procedure volumes, our survey showed that 18% of IRs indicated an increased overall workload with fewer reduction in hours, increased volume of emergency work and less effect on day-case units. This could reflect the increased referrals for IR services in some hospitals.

The initial phases of the pandemic called for increased support and guidance from institutions. Sufficiently available PPE and guidance on the use of PPE were regarded as crucial implementations to ensure staff and patient safety. Especially during the first wave of the pandemic, shortage of PPE was reported, and data were published on dissatisfaction regarding PPE in interventional radiology clinics [3, 14, 15]. Our survey shows that IRs were generally satisfied with the available PPE and PPE guidance, and felt supported by their organisations. Despite that and even with the reduced workload, IRs reported being more stressed and worried about the health of their families and patients, as well as of the general public but not about a potential loss of income or skills. These results could be indicative of increased vulnerability to burnout and anxiety in IRs and other staff as seen in medical personnel of various disciplines [41–44]. Some IRs have voiced concerns about the effect of the reintroduction of pre-COVID routines and the associated workload resulting from the accumulated postponed procedures [45]. Whether the stress and burden of further waves of the pandemic or the increased workload when returning to normal services will have a strong effect on mental health or whether increased adaptation and familiarisation to the needs of the pandemic will prevent that should be closely monitored by the healthcare authorities of each country.

One limitation of our results is the low return-rate of questionnaires despite reminders and efforts for dissemination of the survey. Due to the extensiveness of the survey and since the invitations to participate were sent out during the most intense period of the second wave, the survey was likely not prioritised relative to patient care and safety. The intrinsic limitation of survey-generated data is the vulnerability to discrepancies between the respondents’ perceptions and the results of quantified data. Especially for the second wave of the pandemic, the apex in intensity and infection rates was experienced at different time points in different regions of the world. Additionally, since the data collection for the survey was stopped in March 2021, some effect regarding mental health or the adaptions of IRs might have not been captured in their entirety.

## Conclusion

The results from our survey provide an overview of how the pandemic has affected services and the general workload of IRs. Data indicates that the pandemic resulted in increased stress and concerns but was felt less disruptive during the second wave. Overall, IRs reported to be better prepared for future waves or epidemics. As the pandemic has lasted longer than estimated and is still ongoing, it will be interesting to see how working patterns, workload and mental health will be affected until the end of the pandemic.

## Supplementary Information

Below is the link to the electronic supplementary material.

Supplementary file1 (DOCX 46 KB)

Supplementary file2 (DOCX 21 KB)
