# Supplementary material for: The Impact of COVID-19 on Interventional Radiology Practice Worldwide: Results from a Global Survey
Source: Cardiovasc Intervent Radiol. 2022 Mar 11;45(8):1152–62. doi: 10.1007/s00270-022-03090-6 (PMC8916069; doi:10.1007/s00270-022-03090-6)
Supplement: Supplementary file 1 — Supplementary file1 (DOCX 46 KB) [file 270_2022_3090_MOESM1_ESM.docx]

Supplementary document 1: The effect of COVID 19 on IR services among CIRSE members: questionnaire

Contents

[1. Part 1-Demographic information 5](#_Toc83034303)

[1.1 Gender 5](#_Toc83034304)

[1.2 Location-Country and City 5](#_Toc83034305)

[1.3 Your institution is: 5](#_Toc83034306)

[1.4 Has your IR department cared for Covid-19 positive patients? 5](#_Toc83034307)

[1.4.1 In terms of Covid-19 patient care, when was the most intense period of time during the pandemic? 5](#_Toc83034308)

[1.5 Role in the department 5](#_Toc83034309)

[1.1 Age group 5](#_Toc83034310)

[1.1.1 Was your IR training affected during the pandemic? 6](#_Toc83034311)

[1.1.2 Did you have to be redeployed to cover needs elsewhere in your hospital during the first wave of the pandemic from March to June 2020? 6](#_Toc83034312)

[1.1.3 Did you have to be redeployed to cover needs elsewhere in your hospital during the second wave of the pandemic (from September onwards? 6](#_Toc83034313)

[1.1.4 What is your main worry during the pandemic? 7](#_Toc83034314)

[Please grade each response from 1 to 5 7](#_Toc83034315)

[1.1.5 Did you participate in any webinar IR training before the pandemic 7](#_Toc83034316)

[1.1.6 Did you participate in any webinar IR training during the pandemic 7](#_Toc83034317)

[1.1.7 Did you find the educational webinars useful and would you like them to be continued? 7](#_Toc83034318)

[1.1.8 Do you have access to simulation training in your organization? 7](#_Toc83034319)

[1.1.9 Do you think simulation training in IR procedures would add value to your training? 8](#_Toc83034320)

[1.2 How many IR specialists do you have in your department? 8](#_Toc83034321)

[1.3 How many dedicated personnel (e.g. nurses, technicians, radiographers, medical assistants) do you have in your department? 8](#_Toc83034322)

[1.4 Does you institution normally offer on call (out of hours) IR services 8](#_Toc83034323)

[1.5 Do you have dedicated in-patient beds for the IR department? 8](#_Toc83034324)

[1.5.1 How many 9](#_Toc83034325)

[2. Part 2- Effect on IR services during the pandemic 9](#_Toc83034326)

[General questions 9](#_Toc83034327)

[2.1 Which IR services does your institution offer? 9](#_Toc83034328)

[2.2 Overall, do you think that your workload: 9](#_Toc83034329)

[2.2.1 How was the endovascular aortic work affected in your hospital? 9](#_Toc83034330)

[2.2.2 How was the endovascular peripheral arterial work affected in your hospital? 9](#_Toc83034331)

[2.2.3 How affected was the endourology work in your hospital? 10](#_Toc83034332)

[2.2.4 How affected was the IR hepatobiliary work in your hospital? 10](#_Toc83034333)

[2.2.5 How affected was the interventional oncology work in your hospital? 10](#_Toc83034334)

[2.2.6 How affected was the elective embolization work in your hospital (PAE, UFE)? 10](#_Toc83034335)

[2.3 Are your part of multidisciplinary team meetings? 11](#_Toc83034336)

[2.3.1 How did your multidisciplinary team meeting had to change as a result of the pandemic: 11](#_Toc83034337)

[2.4 How was research activity in your department affected during the pandemic: 11](#_Toc83034338)

[Wave I questions: March to June 2020 12](#_Toc83034339)

[2.5 Did any staff in the IR department have to be redeployed (i.e. moved to a different department or used in a more efficient way) to cover needs elsewhere in your hospital? 12](#_Toc83034340)

[2.5.1 Did you have to be redeployed to cover needs elsewhere in your hospital? 12](#_Toc83034341)

[2.5.2 Did dedicated IR personnel (nurses, technicians, radiographers, medical assistants) in your department have to be redeployed for the needs of COVID? 12](#_Toc83034342)

[2.5.3 Did the above mention redeployments affect the provision of IR services? 12](#_Toc83034343)

[2.6 Did you have to make changes in your working pattern during the Covid-19 pandemic (March-June). (Choose as many as needed) 12](#_Toc83034344)

[2.7 How was the emergency work affected during the first wave of the pandemic? 13](#_Toc83034345)

[2.8 How did the pandemic affect your IR day-case unit in the time period March to June: 13](#_Toc83034346)

[2.9 How did the pandemic affect your IR out patient clinics 13](#_Toc83034347)

[2.9.1 How did you find having your clinics/patient consultations online or over the phone? 14](#_Toc83034348)

[Wave II questions: from September 2020 14](#_Toc83034349)

[2.10 Did any staff in the IR department have to be redeployed to cover needs elsewhere in your hospital during the second wave of the pandemic (from September onwards)? 14](#_Toc83034350)

[2.10.1 Did you have to be redeployed to cover needs elsewhere in your hospital during the second wave of the pandemic (from September onwards)? 14](#_Toc83034351)

[2.10.2 Did dedicated IR personnel (e.g. nurses, technicians, radiographers, medical assistants) in your department have to be redeployed for the needs of COVID during the second wave of the pandemic (from September onwards)? 14](#_Toc83034352)

[2.10.3 Did the above mention redeployments affect the provision of IR services during the second wave of the pandemic (from September onwards)? 15](#_Toc83034353)

[2.11 Did you have to make changes in your working pattern during second wave of the Covid-19 pandemic (from September 2020). (Choose as many as needed) 15](#_Toc83034354)

[2.12 How was the emergency work affected during the second wave of the pandemic? 15](#_Toc83034355)

[2.13 How did the pandemic affect your IR day-case unit in the time period from September onwards: 16](#_Toc83034356)

[2.14 How did the pandemic affect your IR out patient clinics during the second wave of the pandemic (from September onwards)? 16](#_Toc83034357)

[2.14.1 How did you find having your clinics/patient consultations online or over the phone? 16](#_Toc83034358)

[3. Part III- Effect on people and teams 17](#_Toc83034359)

[3.1 Overall did you feel supported by your organization during the most intense periods of the pandemic? 17](#_Toc83034360)

[3.1.1 If not, which aspects could have been/be improved/implemented? (open text answer – Not compulsory) 17](#_Toc83034361)

[3.2 To what extent did you experience anger (e.g. rage, mad, pissed off) during the pandemic? 17](#_Toc83034362)

[3.3 To what extent did you experience fear (e.g. terror, scared, panic) during the the pandemic? 17](#_Toc83034363)

[3.4 To what extent did you experience anxiety (e.g. dread, nervous, worry) during the pandemic? 18](#_Toc83034364)

[3.5 To what extent did you experience sadness (e.g. grief, empty, lonely) during the pandemic? 18](#_Toc83034365)

[3.6 To what extent did you experience relaxation (e.g. calm, easy going, chilled out) during the pandemic? 18](#_Toc83034366)

[3.7 To what extent did you experience happiness (e.g. satisfaction, enjoyment, liking) during the pandemic? 18](#_Toc83034367)

[3.8 What support measures were implemented for you by your institution during the pandemic overall 18](#_Toc83034368)

[3.9 Were you given enough support to work from home: 18](#_Toc83034369)

[3.10 What was your main concern during the intense periods of the pandemic: Please grade each response from 1 to 5 19](#_Toc83034370)

[3.11 Regarding proper guidance on the use of PPEs and performing procedures: 19](#_Toc83034371)

[3.12 Regarding PPEs: 19](#_Toc83034372)

[4. Part IV- Post initial lockdown – service recovery and second wave 20](#_Toc83034373)

[4.1 Have all your services returned to the previous levels of activity? 20](#_Toc83034374)

[4.2 Has your personal day-to-day routine returned to normal? 20](#_Toc83034375)

[4.2.1 If your day to day routine has not returned to normal what is best describing your current routine? (select all that apply) 20](#_Toc83034376)

[4.3 Compared to the period before March 1, 2020, when COVID-19 began spreading across the worlds, do you feel your organization was better prepared for the second wave? 20](#_Toc83034377)

[4.4 Will any of the changes implemented during the pandemic be kept on a long-term basis? 21](#_Toc83034378)

[5. Part VI Industry (we can ask our sponsors for any additional questions) 21](#_Toc83034379)

[5.1 Did you have any Industry support during the pandemic? 21](#_Toc83034380)

[5.2 Did you have access to all the kit you wanted? 21](#_Toc83034381)

[5.3 Did you have access to information and training regarding new devices/techniques? 21](#_Toc83034382)

[5.4 Did you take part in any industry sponsored education/training webinars during the pandemic? 22](#_Toc83034383)

[5.5 Would you be open to virtual consultation with industry reps when doing cases to avoid unnecessary travel? 22](#_Toc83034384)

# Part 1-Demographic information

## Gender

Male

Female

Other

## Location-Country and City

## Your institution is:

- Tertiary centre

- Public district general hospital (>500 beds)

- Public district general hospital (<500 beds)

- Public district hospital (>500 beds) (<500 beds)

- Public district general hospital

-Other

## Has your IR department cared for Covid-19 positive patients?

- Yes
- No
- Don’t want to say
- Don’t know

*If yes*

### In terms of Covid-19 patient care, when was the most intense period of time during the pandemic?

- First wave, i.e. March to June 2020
- Second wave, i.e. since September 2020
- Both waves are intense
- I am unsure

## Role in the department

- Resident
- IR fellow in training
- Completed IR training/specialist
- Board certified radiologist
- Other

## Age group

- <35
- 35-45
- 46-55
- 56+

*If Trainee:*

### Was your IR training affected during the pandemic?

- No, not at all
- Mostly not
- Yes but to a small degree
- Yes and in a significant degree
- I don’t know

*If -Yes but to small degree*

*-Yes and in a significant degree*

#### What do you think was the main reason? (select all that apply)

-Lack of organisation from the hospital

-Changes in the team due to redeployment (i.e. moving staff to a different department or using them in a more effective way)

-Lack of proper PPEs (i.e. personal protective equipment

-Bulk of work compared to a normal situation

-All the above

Other

### Did you have to be redeployed to cover needs elsewhere in your hospital during the first wave of the pandemic from March to June 2020?

-Yes, for the duration of the first wave

-Yes, for a couple of weeks

-Yes, for some shifts

-No

-I am unsure

### Did you have to be redeployed to cover needs elsewhere in your hospital during the second wave of the pandemic (from September onwards?

-Yes, for the duration of the second wave of the pandemic

-Yes, for a couple of weeks

-Yes, for some shifts

-No

-I am unsure

### What is your main worry during the pandemic?

## Please grade each response from 1 to 5

| 1 | 2 | 3 | 4 | 5 |
| --- | --- | --- | --- | --- |
| Not at all worried | Slightly worried | Somewhat worried | Moderately worried | Extremely worried |

-How my training is going to be affected

- How the job market is going to be affected.

-Travel restrictions affecting opportunities for fellowships abroad.

-Other

### Did you participate in any webinar IR training before the pandemic

-No

-Yes once a week

-Yes once every 2 weeks

-Yes once every month

- Other

### Did you participate in any webinar IR training during the pandemic

-No

-Yes once a week

-Yes once every 2 weeks

-Yes once every month

-Other

### Did you find the educational webinars useful and would you like them to be continued?

-Yes prefer them to face-to-face meetings

-No prefer the face-to-face meetings

-I would like to see a combination of both face to face and virtual meetings in the future

-I don’t know

### Do you have access to simulation training in your organization?

-No access

-Limited access /shared access with other specialty

-Full access

-I don’t know

### Do you think simulation training in IR procedures would add value to your training?

-No

-Somewhat improbable

-Somewhat probable, but to small degree

-Very probable and to a significant degree

-I don’t know

## How many IR specialists do you have in your department?

- <5

- 5-10

- 10+

## How many dedicated personnel (e.g. nurses, technicians, radiographers, medical assistants) do you have in your department?

- <5

- 5-10

- 10+

## Does you institution normally offer on call (out of hours) IR services

-Yes 24/7

-Yes but limited (not 24/7)

-No on call

-I am unsure

## Do you have dedicated in-patient beds for the IR department?

Yes

No

-I am unsure

If yes

### How many

# Part 2- Effect on IR services during the pandemic

## General questions

## Which IR services does your institution offer?

- endovascular aortic services

- endovascular peripheral arterial services

- endourology services

- hepatobiliary services

- interventional oncology

- elective embolization

## Overall, do you think that your workload:

-Increased a lot

- Mildly Increased

- Remained stable

- Mildly decreased

- Decreased a lot

-I am unsure

*If service selected to be offered at hospital above:*

### How was the endovascular aortic work affected in your hospital?

- Elective and urgent cases performed as normal

- Only emergencies were performed-all non-urgent cases cancelled

- Case by case discussion and decision

-Completely or almost completely stopped

-I am unsure

-Other

### How was the endovascular peripheral arterial work affected in your hospital?

- Elective and urgent cases performed as normal

- Only emergencies were performed-all non-urgent cases cancelled

- Case by case discussion and decision

-Completely or almost completely stopped

-I am unsure

-Other

### How affected was the endourology work in your hospital?

- Elective and urgent cases performed as normal

- Only emergencies were performed-all non-urgent cases cancelled

- Case by case discussion and decision

- Completely or almost completely stopped

-I am unsure

-Other

### How affected was the IR hepatobiliary work in your hospital?

- Elective and urgent cases performed as normal

- Only emergencies were performed-all non-urgent cases cancelled

- Case by case discussion and decision

-Completely or almost completely stopped

--I am unsure

-Other

### How affected was the interventional oncology work in your hospital?

- Elective and urgent cases performed as normal

- Only emergencies were performed-all non-urgent cases cancelled

- Case by case discussion and decision

-Completely or almost completely stopped

-I am unsure

-Other

### How affected was the elective embolization work in your hospital (PAE, UFE)?

- Elective and urgent cases performed as normal

- Only emergencies were performed-all non-urgent cases cancelled

- Case by case discussion and decision

-Completely or almost completely stopped

-I am unsure

-Other

## Are your part of multidisciplinary team meetings?

- Yes

- No

*If yes:*

### How did your multidisciplinary team meeting had to change as a result of the pandemic:

-No change

- Number of participants reduced in face to face meeting

-Some of them performed virtually and the rest face to face

-All of them performed virtually

-Had to be stopped completely

-Other

*If All of them performed virtually*

*-Some of them performed virtually and the rest face to face*

#### How did you find having multidisciplinary meetings online?

-Excellent, worked perfectly

- Good, worked well most of the time

- Fair, worked well some of the times

- Poor, there were a lot of issues

-Very poor, it never really worked

-I am unsure

## How was research activity in your department affected during the pandemic:

- Due to the reduction in clinical work we had a lot more time for research activities.

- No effect, all projects continued as before

- Mildly affected, some projects were delayed or halted

- Severely affected, most or all projects were delayed or halted

- I am unsure

## Wave I questions: March to June 2020

## Did any staff in the IR department have to be redeployed (i.e. moved to a different department or used in a more efficient way) to cover needs elsewhere in your hospital?

- Yes, most of them

- Yes, a few

- No

-I am unsure

*If yes:*

### Did you have to be redeployed to cover needs elsewhere in your hospital?

-Yes, for the duration of the first wave of the pandemic from March –June 2020

-Yes, for a couple of weeks

-Yes, for some shifts

-No

-I am unsure

### Did dedicated IR personnel (nurses, technicians, radiographers, medical assistants) in your department have to be redeployed for the needs of COVID?

-No redeployment

-Only few of them were deployed

-Most of them were redeployed

-I am unsure

### Did the above mention redeployments affect the provision of IR services?

-No, not at all.

-Some services were delayed or halted

-Most services were delayed or halted

- All services were delayed or halted

-I am unsure

## Did you have to make changes in your working pattern during the Covid-19 pandemic (March-June). (Choose as many as needed)

- Segregated working teams to reduce number of people in department

- Consolidating working hours to reduce number of people in the department

- Consolidating working hours because of fewer referrals

- Working from home

- Reducing hours at the hospital

- Reducing operating lists

- Other

## How was the emergency work affected during the first wave of the pandemic?

-Significantly increased volume

-Increased volume

- Unchanged volume

-Decreased volume

-Significantly decreased volume

-I am unsure

## How did the pandemic affect your IR day-case unit in the time period March to June:

- Strongly affected - the unit had to close for the entire time March to June.

- Affected, the unit had to close for a part of the time March to June.

- No change to the service.

-Not affected, the unit stayed open but could only receive reduced patient numbers.

-Not at all affected, the unit stayed open and patient numbers increased.

-Other

-Not applicable.

## How did the pandemic affect your IR out patient clinics

- Strongly affected -had to be cancelled.

- Affected - had to be done virtually (webconference or over the phone)

- No change to the service.

-Not affected, performed reduced face to face

-Not at all affected, Performed face to face as normal.

-Other

-Not applicable.

*If* -*Had to be done virtually (webconference with patient)*

### How did you find having your clinics/patient consultations online or over the phone?

- Excellent - worked well all of the time

- Good - worked well most of the times

- Fair – worked well some of the times

- Poor – did not work well most of the time

- Very poor - never really worked

- I am unsure

Comment [open text

## Wave II questions: from September 2020

## Did any staff in the IR department have to be redeployed to cover needs elsewhere in your hospital during the second wave of the pandemic (from September onwards)?

- Yes, most of them

- Yes, a few

- No

-I am unsure

*If yes:*

### Did you have to be redeployed to cover needs elsewhere in your hospital during the second wave of the pandemic (from September onwards)?

-Yes, for the duration of the first wave of the pandemic from March –June 2020

-Yes, for a couple of weeks

-Yes, for some shifts

-No

-I am unsure

### Did dedicated IR personnel (e.g. nurses, technicians, radiographers, medical assistants) in your department have to be redeployed for the needs of COVID during the second wave of the pandemic (from September onwards)?

-No redeployment

-Only few of them were deployed

-Most of them were redeployed

-I am unsure

### Did the above mention redeployments affect the provision of IR services during the second wave of the pandemic (from September onwards)?

-No, not at all.

-Some services were delayed or halted

-Most services were delayed or halted

- All services were delayed or halted

-I am unsure

## Did you have to make changes in your working pattern during second wave of the Covid-19 pandemic (from September 2020). (Choose as many as needed)

- Segregated working teams to reduce number of people in department

- Consolidating working hours to reduce number of people in the department

- Consolidating working hours because of fewer referrals

- Working from home

- Reducing hours at the hospital

- Reducing operating lists

- Other

## How was the emergency work affected during the second wave of the pandemic?

-Significantly increased volume

-Increased volume

- Unchanged volume

-Decreased volume

-Significantly decreased volume

-I am unsure

## How did the pandemic affect your IR day-case unit in the time period from September onwards:

- Strongly affected - the unit had to close for the entire time March to June.

- Affected, the unit had to close for a part of the time March to June.

- No change to the service.

-Not affected, the unit stayed open but could only receive reduced patient numbers.

-Not at all affected, the unit stayed open and patient numbers increased.

-Other

-Not applicable.

## How did the pandemic affect your IR out patient clinics during the second wave of the pandemic (from September onwards)?

- Strongly affected -had to be cancelled.

- Affected - had to be done virtually (webconference or over the phone)

- No change to the service.

-Not affected, performed reduced face to face

-Not at all affected, Performed face to face as normal.

-Other

-Not applicable.

*If* -*Had to be done virtually (webconference with patient)*

### How did you find having your clinics/patient consultations online or over the phone?

- Excellent - worked well all of the time

- Good - worked well most of the times

- Fair – worked well some of the times

- Poor – did not work well most of the time

- Very poor - never really worked

- I am unsure

# Part III- Effect on people and teams

## Overall did you feel supported by your organization during the most intense periods of the pandemic?

-Not at all

-Slightly

- Somewhat

-Quite a bit

-Very much-

-I am unsure

*If no:*

### If not, which aspects could have been/be improved/implemented? (open text answer – Not compulsory)

3. Did you feel the pandemic increased your stress levels at work?

-Not at all

-Slightly

- Somewhat

-Quite a bit

-Very much

-I am unsure

## To what extent did you experience anger (e.g. rage, mad, pissed off) during the pandemic?

| 1 | 2 | 3 | 4 | 5 | 6 | 7 |
| --- | --- | --- | --- | --- | --- | --- |
| Not at all | Slightly | Somewhat | Moderately | Quite a bit | Very much | An extreme amount |

## To what extent did you experience fear (e.g. terror, scared, panic) during the the pandemic?

| 1 | 2 | 3 | 4 | 5 | 6 | 7 |
| --- | --- | --- | --- | --- | --- | --- |
| Not at all | Slightly | Somewhat | Moderately | Quite a bit | Very much | An extreme amount |

## To what extent did you experience anxiety (e.g. dread, nervous, worry) during the pandemic?

| 1 | 2 | 3 | 4 | 5 | 6 | 7 |
| --- | --- | --- | --- | --- | --- | --- |
| Not at all | Slightly | Somewhat | Moderately | Quite a bit | Very much | An extreme amount |

## To what extent did you experience sadness (e.g. grief, empty, lonely) during the pandemic?

| 1 | 2 | 3 | 4 | 5 | 6 | 7 |
| --- | --- | --- | --- | --- | --- | --- |
| Not at all | Slightly | Somewhat | Moderately | Quite a bit | Very much | An extreme amount |

## To what extent did you experience relaxation (e.g. calm, easy going, chilled out) during the pandemic?

| 1 | 2 | 3 | 4 | 5 | 6 | 7 |
| --- | --- | --- | --- | --- | --- | --- |
| Not at all | Slightly | Somewhat | Moderately | Quite a bit | Very much | An extreme amount |

## To what extent did you experience happiness (e.g. satisfaction, enjoyment, liking) during the pandemic?

| 1 | 2 | 3 | 4 | 5 | 6 | 7 |
| --- | --- | --- | --- | --- | --- | --- |
| Not at all | Slightly | Somewhat | Moderately | Quite a bit | Very much | An extreme amount |

## What support measures were implemented for you by your institution during the pandemic overall

- Support for my mental health and wellbeing

- Support for working from home

- Support for prolonged stays at the hospital/work hours

- Other support provided

- No specific support was provided

## Were you given enough support to work from home:

-Yes, Had all the support I needed

-Somewhat, I had some support

-No support

-

-

-No option for home working was provided.

## What was your main concern during the intense periods of the pandemic: Please grade each response from 1 to 5

| 1 | 2 | 3 | 4 | 5 |
| --- | --- | --- | --- | --- |
| Not at all concerned | Slightly concerned | Somewhat concerned | Moderately concerned | Extremely concerned |

-The health of my family and me

-The health of my patients

- Public health as a whole/development of the pandemic

-Loss of skills

-Loss of income

-No concerns

-Other

## Regarding proper guidance on the use of PPEs and performing procedures:

-Completely satisfied, I had enough support and guidelines from my department/institution

-Generally satisfied, I had enough support but it could have been better

-Somewhat dissatisfied, I didn’t have enough support and guidelines from my department/institution

-Completely dissatisfied, I didn’t have support at all

-

-Other (please comment)

## Regarding PPEs:

- Completely satisfied, I always had enough PPEs to perform my duties

-Generally satisfied, there were times we didn’t have enough PPEs to perform our duties

-Somewhat dissatisfied, there was usually a lack of proper PPEs.

-Completely dissatisfied, no proper PPEs at all

-I don’t know/Other

# Part IV- Post initial lockdown – service recovery and second wave

## Have all your services returned to the previous levels of activity?

-No

-Almost back to normal

-Back to normal

-Back to normal, but with provisions for the second wave

-I don’t know

## Has your personal day-to-day routine returned to normal?

- Completely
- Mostly yes
- Somewhat
- Mostly not
- Not at all

### If your day to day routine has not returned to normal what is best describing your current routine? (select all that apply)

-Still able to work from home

-Many/most meetings still taking place virtually

-Segregate teams and adjusted job plans to prevent to many people at work

-Still reduced IR cases/referrals

-Back to pre-covid routine

-I don’t know

-Other (please tell us)

## Compared to the period before March 1, 2020, when COVID-19 began spreading across the worlds, do you feel your organization was better prepared for the second wave?

-No

-Somewhat better, but to a small degree

-Much better and to a significant degree

-Yes

-I don’t know

## Will any of the changes implemented during the pandemic be kept on a long-term basis?

-No

-Yes

-Potentially

-To be decided

-I don’t know

If yes, please state which ones (open text answer)

# Part VI Industry (we can ask our sponsors for any additional questions)

## Did you have any Industry support during the pandemic?

-No

-Very limited

-Some support

-All the support I needed

## Did you have access to all the kit you wanted?

-Never, always some delay and/or issues with availability of supplies

-Rarely, very often delays and/or issues with availability of supplies

-Sometimes, some delays and/or issues with availability of supplies

-Often, almost no delays and/or issues with availability of supplies

-Always, no delays or issues with availability of supplies

## Did you have access to information and training regarding new devices/techniques?

-No access

-Limited access

-Good access

-I don’t know

## Did you take part in any industry sponsored education/training webinars during the pandemic?

-No

-Yes once in a week

-Yes once every 2 weeks

- Yes once every months

-I don’t know

## Would you be open to virtual consultation with industry reps when doing cases to avoid unnecessary travel?

-Yes if there was a good platform

-No prefer face-to-face consultation during cases

-I Don’t know

-Other
