# Supplementary material for: The Impact of COVID-19 on Interventional Radiology Practice Worldwide: Results from a Global Survey
Source: Cardiovasc Intervent Radiol. 2022 Mar 11;45(8):1152–62. doi: 10.1007/s00270-022-03090-6 (PMC8916069; doi:10.1007/s00270-022-03090-6)
Supplement: Supplementary file 2 — Supplementary file2 (DOCX 21 KB) [file 270_2022_3090_MOESM2_ESM.docx]

**Supplementary document 2**: Differences in working patterns, stress levels and support received by the organization for respondents that indicated “increased” or “mildly increased” compared to respondents that indicated “remained stable”, “decreased a lot” or “mildly decreased” overall workload. Significant differences between categorical variables were assessed using Fisher exact test.

|  | Overall workload increased or mildly increased | | Overall workload remained stable, decreased a lot, or mildly decreased | | P value |
| --- | --- | --- | --- | --- | --- |
|  | **n** | **%** | **n** | **%** | **P value** |
| **Type of institution** |  |  |  |  |  |
| Tertiary centre | 20 | 44 | 103 | 47 | n.s.  p=0.68 |
| Public district hospital (>500 beds) | 13 | 29 | 43 | 20 |  |
| Public district hospital (<500 beds) | 3 | 7 | 19 | 9 |  |
| Private hospital | 8 | 18 | 40 | 18 |  |
|  |  |  |  |  |  |
| **Change in working patters – first wave** |  |  |  |  |  |
| Segregated working teams to reduce number of people in department | 28 | 62 | 107 | 49 |  |
| Consolidating working hours to reduce number of people in the department | 16 | 36 | 61 | 28 |  |
| Consolidating working hours because of fewer referrals | 4 | 9 | 44 | 20 |  |
| Working from home | 9 | 20 | 54 | 25 |  |
| Reducing hours at the hospital | **3** | **7** | **68** | **31** | p<0.001 |
| Reducing operating lists | 17 | 38 | 96 | 44 |  |
|  |  |  |  |  |  |
| **Emergency work – first wave** |  |  |  |  |  |
| Significantly increased volume | 4 | 9 | 9 | 4 | p<0.001 |
| Increased volume | **18** | **40** | **13** | **6** |  |
| Unchanged volume | **10** | **22** | **60** | **27** |  |
| Decreased volume | **11** | **24** | **105** | **48** |  |
| Significantly decreased volume | **2** | **4** | **29** | **13** |  |
|  |  |  |  |  |  |
| **Day case – first wave** |  |  |  |  |  |
| Strongly affected - the unit had to close for the entire time March to June. | 1 | 2 | 12 | 5 | p=0.006 |
| Affected, the unit had to close for a part of the time March to June. | **10** | **22** | **55** | **25** |  |
| No change to the service. | **12** | **27** | **42** | **19** |  |
| Not affected, the unit stayed open but could only receive reduced patient numbers. | **13** | **29** | **96** | **44** |  |
| Not at all affected, the unit stayed open and patient numbers increased. | **8** | **18** | **4** | **2** |  |
|  |  |  |  |  |  |
| **Out patient – first wave** |  |  |  |  |  |
| Strongly affected -had to be cancelled. | 10 | 22 | 67 | 30 | n.s.  p=0.13 |
| Affected - had to be done virtually (webconference or over the phone) | 9 | 20 | 57 | 26 |  |
| No change to the service. | 9 | 20 | 37 | 17 |  |
| Not affected, performed reduced face to face | 12 | 27 | 43 | 20 |  |
| Not at all affected, Performed face to face as normal. | 4 | 9 | 5 | 2 |  |
|  |  |  |  |  |  |
| **Change in working patters – second wave** |  |  |  |  |  |
| Segregated working teams to reduce number of people in department | 12 | 27 | 48 | 22 |  |
| **Consolidating working hours to reduce number of people in the department** | **19** | **42** | **43** | **20** | **p=0.002** |
| Consolidating working hours because of fewer referrals | 5 | 11 | 35 | 16 |  |
| Working from home | 6 | 13 | 36 | 16 |  |
| Reducing hours at the hospital | **2** | **4** | **42** | **19** | **p=0.015** |
| Reducing operating lists | 9 | 20 | 79 | 36 |  |
|  |  |  |  |  |  |
| **Emergency work – second wave** |  |  |  |  |  |
| Significantly increased volume | **11** | **24** | **7** | **3** | p<0.001 |
| Increased volume | **14** | **31** | **24** | **11** |  |
| Unchanged volume | **16** | **36** | **102** | **46** |  |
| Decreased volume | **4** | **9** | **72** | **33** |  |
| Significantly decreased volume | 0 | 0 | 10 | 5 |  |
|  |  |  |  |  |  |
| **Day case – second wave** |  |  |  |  |  |
| Strongly affected - the unit had to close for the entire time March to June. | 1 | 2 | 9 | 4 | p=0.006 |
| Affected, the unit had to close for a part of the time March to June. | 8 | 18 | 29 | 13 |  |
| No change to the service. | 17 | 38 | 84 | 38 |  |
| Not affected, the unit stayed open but could only receive reduced patient numbers. | **10** | **22** | **74** | **34** |  |
| Not at all affected, the unit stayed open and patient numbers increased. | 9 | 20 | 8 | 4 |  |
|  |  |  |  |  |  |
| **Out patient – second wave** |  |  |  |  |  |
| Strongly affected -had to be cancelled. | 2 | 4 | 27 | 12 | n.s.  p=0.12 |
| Affected - had to be done virtually (webconference or over the phone) | 5 | 11 | 37 | 17 |  |
| No change to the service. | 24 | 53 | 73 | 33 |  |
| Not affected, performed reduced face to face | 10 | 22 | 48 | 22 |  |
| Not at all affected, Performed face to face as normal. | 4 | 9 | 10 | 5 |  |
|  |  |  |  |  |  |
| **Supported by organization** |  |  |  |  |  |
| Not at all | 3 | 7 | 11 | 5 | n.s.  p=0.65 |
| Slightly | 9 | 20 | 27 | 12 |  |
| Somewhat | 11 | 24 | 58 | 26 |  |
| Quite a bit | 10 | 22 | 61 | 28 |  |
| Very much | 12 | 27 | 61 | 28 |  |
|  |  |  |  |  |  |
| **Stress levels at work** |  |  |  |  |  |
| Not at all | 1 | 2 | 22 | 10 | n.s.  p=0.46 |
| Slightly | 6 | 13 | 31 | 14 |  |
| Somewhat | 10 | 22 | 51 | 23 |  |
| Quite a bit | 12 | 27 | 57 | 26 |  |
| Very much | 16 | 36 | 59 | 27 |  |
